# Upregulation of miR-1825 inhibits the progression of glioblastoma by suppressing CDK14 though Wnt/β-catenin signaling pathway

**DOI:** 10.1186/s12957-020-01927-3

**Published:** 2020-06-30

**Authors:** Fengqin Lu, Chunhong Li, Yuping Sun, Ting Jia, Na Li, Haiyan Li

**Affiliations:** 1 Department of Geriatrics, Jinan Zhangqiu District Hospital of TCM, Jinan, 250200 China; 2 Department of Public Health, Jinan Zhangqiu District Hospital of TCM, Jinan, 250200 China; 3 Department of Neurology, the People’s Hospital of Zhangqiu Area, Jinan, 250200 China; 4 Department of Gynaecology, the People’s Hospital of Zhangqiu Area, Jinan, 250200 China; 5 Department of Radiology, the People’s Hospital of Zhangqiu Area, Jinan, 250200 China; 6 Department of Neurology, Qingdao Central Hospital, Qingdao University, No.127 Siliunan Road, Qingdao, 266042 China

**Keywords:** miR-1825, CDK14, Glioblastoma, Wnt/β-catenin

## Abstract

**Background:**

Mounting evidences displayed that miRNAs play crucial roles in tumor initiation and development. However, the regulation and relevant mechanism of miR-1825 in glioblastoma (GBM) remain unclear.

**Methods:**

qRT-PCR was used to detect miR-1825 and CDK14 mRNA expression. Western blot was applied for testing protein levels (VEGF, E-cadherin, N-cadherin, vimentin, β-catenin, c-myc, p-c-Jun). MTT and transwell assays were used for detecting GBM cell progression, including cell viability, migration, and invasion.

**Results:**

The results showed that miR-1825 was decreased in GBM tissue specimens by qRT-PCR and it was confirmed as a prognostic marker of GBM by Kaplan-Meier survival analysis. Moreover, we also found that miR-1825 upregulation suppressed GBM cell viability, tumor growth, invasion, and migration. Furthermore, CDK14 was first identified as the direct target of miR-1825 by Luciferase reporter assay. CDK14 acted as an oncogene in GBM development by immunohistochemistry. In addition, Western blot analysis demonstrated that miR-1825 regulated Wnt/β-catenin signaling pathway in GBM development.

**Conclusion:**

In conclusion, miR-1825 upregulation suppressed GBM progression by targeting CDK14 through Wnt/β-catenin pathway.

## Introduction

Glioblastoma (GBM) are tumors derived from neuroepithelial tumors, accounting for 40 to 50% of brain tumors [1, 2]. They are the most common intracranial malignant tumors, which are caused by the interaction of congenital genetic high-risk factors and environmental carcinogenic factors [3, 4]. Although the current treatment for GBM patients has made great improvement, the survival rate of GBM patients was still very poor. Moreover, the high recurrence of GBM makes it very difficult to cure. Thus, it is very urgent to understand the underlying mechanism of GBM and to explore novel targets for GBM treatment.

Currently, miRNAs were determined as the potential in cancer treatment [5, 6] and it can modulate a tumor’s development and progression by regulating their target genes [7–9]. In GBM, many miRNAs have been proved to participate in cell progression and development. For instance, miR-365 displayed the inhibitory effect on GBM cell viability and migration via targeting PAX6 [10]. Besides, miR-210 was proved to inhibit GBM cell invasion and migration by targeting BNDF [11]. Moreover, the findings of Cui T et al displayed that miR-4516 can target PTPN14 to promote GBM cell progression and miR-4516/ PTPN14 axis provided an insight for treating GBM [12]. However, the researches of miR-1825 in GBM development and progression are very little. Xing and his colleagues showed that the expression of miR-1825 was decreased in glioma and it could suppress cell proliferation and invasion and facilitate cell apoptosis of glioma cell [13]. These findings suggested the possibility that miR-1825 might have an important role in regulating in GBM development and progression.

Cyclin-dependent kinases (CDKs), which are critical regulatory enzymes that drive cell cycle transition, are serine/threonine kinases characterized by their need for a separate subunit a cyclin—to provide the essential domains for the enzymatic activity of the CDKs [14, 15]. CDK14, also called PFTK1 (PFTAIRE protein kinase 1), is well known to regulate cell cycle and play important roles in cellular activities [16]. As previous studies displayed, CDK14 took part in multiple cancers’ development as a target of miRNAs [17]. For example, it was the target of miR-542 and involved in ovarian cancer cell proliferation, invasion, and tumorigenesis [18]. Also, CDK14 acted as the target of miR-431 in regulating pancreas cancer development [19]. Furthermore, CDK14 functioned as an oncogene in glioma and served as a target gene of miR-613 [20]. Based on the studies above, we want to verify whether CDK14 is the target of miR-1825 in regulating GBM progression, which has not been reported until now.

Epithelial-mesenchymal transition (EMT) is an important biological process for the migration and invasion of epithelial-derived malignant cells [21]. The activation of Wnt/β-catenin signaling pathway promotes tumor progression [22]. Therefore, we further investigated miR-1825 effect on EMT and Wnt/β-catenin axis. Collectively, the goal of this study was to investigate the role of miR-1825 in GBM progression and explore whether miR-1825 inhibited cell proliferation, invasion, and migration by regulating CDK14 and Wnt/β-catenin signaling pathway.

## Materials and methods

### GBM tissues

Fifty-five paired fresh tissue specimens were collected from GBM patients who were recruited between March 2013 and September 2017 at Jinan Zhangqiu District Hospital of TCM. All GBM patients have not received any treatment before surgery. The fresh tissues were verified by pathologists and stored at a − 80 °C refrigerator for further use. All patients have provided written consent to allow for research purposes prior to the collection of tissue samples. The histological features of all specimens were confirmed by pathologists according to the WHO criteria. The research was approved by the ethic committee of Jinan Zhangqiu District Hospital of TCM.

### Cell culture

GBM cell lines U251, U87, and A172 were obtained from BeNa Culture Collection (Suzhou, China). Normal human astrocytes (NHA) were purchased from BeNa Culture Collection (Suzhou, China). All the cells were cultured as previously described [23]. Then the cells were maintained in a humidified incubator containing 5% CO_2_ at 37 °C.

### MiR-1825 mimic and miR-1825 inhibitor

The mimic or inhibitor of miR-1825 was purchased from Shanghai GenePharma Co., Ltd. (Shanghai, China). They were used for increasing or decreasing the expression of miR-1825. A172 cells were the selected cells for further analysis. The transfection was conducted for 48 h following the instructions of Lipofectamine 2000 reagent (Invitrogen, Thermo Fisher Scientific).

### Real-time PCR (RT-PCR)

The mRNA expression of miR-1825 and CDK14 was measured by RT-PCR. Total RNAs were firstly isolated from GBM tissues and cells using TRIzol reagent (Invitrogen) for both mRNA and miRNA analyses. The miScript Reverse Transcription kit (Beyotime, Haimen, Jiangsu, China) was carried out for producing cDNA. Then, quantitative PCR was conducted by a miScript SYBR-Green PCR kit (Beyotime) at an ABI 7500 Real-time PCR system (Applied Biosystems, Thermo Fisher Scientific). U6 was applied for normalizing miR-1825 relative mRNA expression and GAPDH for normalizing CDK14. ^2−ΔΔCq^ method was used for calculating the gene expression. The primers are shown in supplemental table 1.

### Western blot

The relative protein level was tested by Western blot. In brief, the total proteins were exacted from GBM cells using RIPA lysis buffer and then conducted the protein concentration by BCA kit (Beyotime). After the equal protein separated by 10% SDS-PAGE, they were transferred to the NC membranes. Then, the membranes were blocked with 5% skimmed milk powder at 37 °C for 1 h, incubated with primary antibodies (CDK14, ab167928, 1:1,000; E-cadherin, ab76055, 1:1,000; N-cadherin, ab18203, 1:1,000; Vimentin, ab92547, 1:1,000; β-catenin, ab32572, 1:1,000; c-myc, ab32072, 1:1,000; p-c-Jun, ab32385, 1:1,000; GAPDH, ab181602, 1:1,000; all from Abcam) at 4 °C overnight, and secondary antibodies at 37 °C for 1 h. Finally, an enhanced chemiluminescence (ECL) method and the ImageJ software were applied for detecting the immune complexes and quantified protein levels, respectively.

### Cell proliferation analysis

Cell proliferation was evaluated by 3- (4, 5-dimethylthiazolyl-2)- 2,5-diphenyltetrazolium bromide (MTT) assay. Briefly, A172 cells were placed in 96-well plates at a density of 2000 cells/well. When the cells were cultured for 1, 2, 3, and 4 days, MTT solution (20 μl) was added to each well and incubated for another 4 h at 37 °C. Then, the medium was removed and dimethyl sulfoxide (DMSO) was added to dissolve formazan crystals by swirling gently. Finally, the optical density was detected at a wavelength of 490 nm using a microplate reader.

### Cell migration and invasion analysis

The invasiveness and metastasis of U87 cells were measured by Transwell assay. Detection of invasion and migration was similar, except for the upper chamber coated with Matrigel. Briefly, the cells were added to the upper chamber, and DMEM containing 20% FBS was added to the lower chamber. After incubation for 24 h, the cells that traversed the membrane were fixed, stained, and counted. These traversed cells were used to evaluate cell invasion and migration.

### Immunohistochemistry analysis

Immunohistochemistry was applied for detecting CDK14 protein density. Firstly, the paraffin sections were obtained from GBM tissues. Then they were incubated with 3% H_2_O_2_ for 15 min. After blocking with goat serum for 2 h at room temperature, the sections were incubated with primary antibodies at 4 °C overnight and secondary antibodies at 37 °C for 1 h. Next, the sections were stained using DAB solution, followed by alcohol dehydration, xylene decolorization, and neutral resin sealing. Finally, the results were observed and photographed by a microscope.

### Tumor growth analysis

Xenograft tumor formation assays were applied for measuring the tumor growth in vivo. The Animal Research Committee of Jinan Zhangqiu District Hospital of TCM approved these animal experiments. The operation meets the standards for laboratory animal care and use in Jinan Zhangqiu District Hospital of TCM. A172 cells (1 × 10^5^) treated with miR-1825 mimic or NC were injected into right flank of nude mice subcutaneously, which were purchased from Shanghai Laboratory Animal Center (Shanghai, China). Then, the tumor size and weight was measured every 4 days for 28 days by a vernier caliper and electronic scale, respectively. All mouse experiments were carried out in accordance with institutional guidelines and regulations of the government.

### Luciferase activity analysis

Bioinformatic analysis algorithm TargetScan (http://www.targetscan.org/vert_72/) was used to predict the targets of miR-1825. The binding site of miR-1825 in the 3′-untranslated region (3′-UTR) of CDK14 (wild-type or mutant) was cloned into pGL3-reporter vector (Promega, Madison, WI, USA). Then, A172 cells were co-transfected with the NC vector and miR-1825 mimic using Lipofectamine 2000 (Invitrogen). After transfection for 48 h, the luciferase activity was tested by the Dual-Luciferase Reporter Assay System (Promega), and *Renilla* luciferase activity was used to normalize the data.

### Statistics analysis

The values were represented as mean ± SD. All experimental conditions were repeated in duplicate independently. Data was analyzed by SPSS 22.0 statistical software (SPSS, Inc.) and the statistics was performed by GraphPad Prism 6. Student’s *t* test was applied for comparing the difference between two groups, and Tukey’s post hoc test of one-way analysis of variance (one-way ANOVA) was carried out for comparing the differences between more than two groups. The Pearson test was applied for determining the relationship between miR-1825 and CDK14. Log-rank test was applied for analyzing the survival rate. *P* < 0.05 was considered statistically significant.

## Results

### MiR-1825 downregulation was associated with poor prognosis

**Fig. 1:**
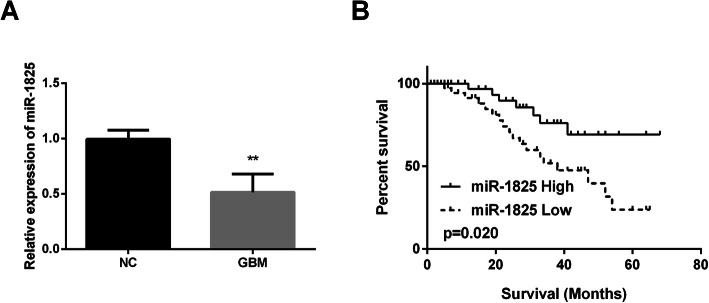
The association of miR-1825 differential expression with overall survival in GBM. **a** Decreased expression of miR-1825 in GBM tissue samples (*n* = 55). **b** Higher expression of miR-1825 in GBM patients exhibited a higher survival rate of GBM patients. **P < 0.01

Here, we detected miR-1825 expression in GBM tissue specimens. The findings displayed that expression of miR-1825 was downregulated in GBM tissues compared with that in normal tissues (Fig. 1a). Moreover, we investigated whether the differential expression of miR-1825 was related to patients’ survival rate. The results of Fig. 1b display that GBM with high expression of miR-1825 predicted better prognosis, while low expression predicted poorer prognosis. Moreover, miR-1825 was significantly associated with GBM clinicopathological features, including WHO grade (Table 1). These findings demonstrated that miR-1825 downregulation served as an indicator for poorer prognosis of GBM patients.

**Table 1:** The clinicopathological relevance analysis of miR-1825 expression in GBM patients

Characteristics	Cases	miR-1825		*P* value
		High	Low	
**Age (years)**				0.761
≥ 60	25	9	16	
< 60	30	12	18	
**Gender**				0.399
Male	35	12	23	
Female	20	10	12	
**WHO grade**				0.022*
I + II	20	12	8	
III + IV	35	10	25	
**Location**				0.425
Supratentorial	30	14	16	
Subtentorial	25	9	16	

Statistical analyses were performed by the *χ*^2^ test

*P < 0.05 was considered significant

### MiR-1825 suppressed GBM progression

**Fig. 2:**
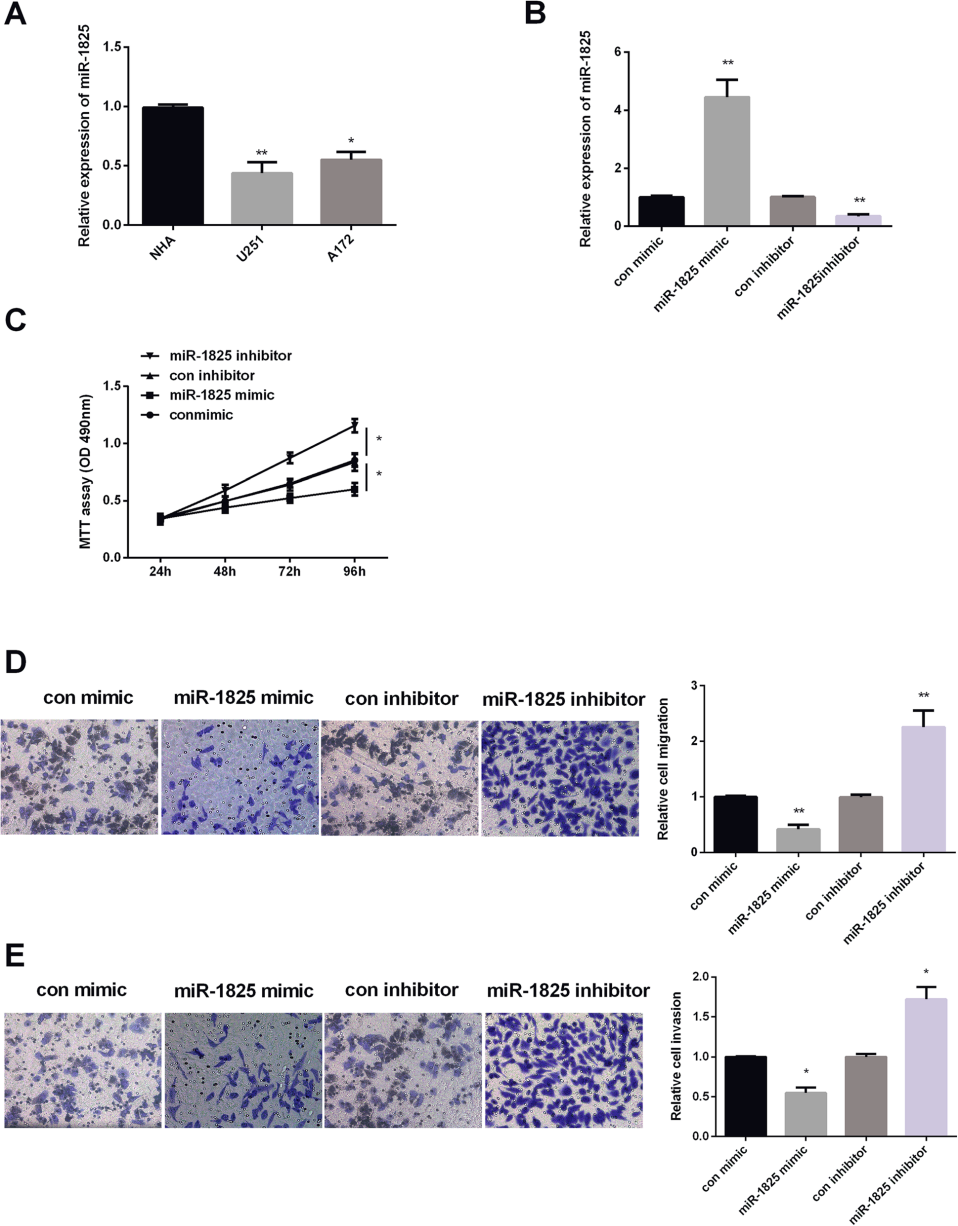
Hindrance effect of miR-1825 on GBM progression. **a** Decreased expression of miR-1825 in GBM cell lines. **b** Decreased miR-1825 expression in miR-1825 inhibitor group and increased miR-1825 expression in miR-1825 mimic group in A172 cells. **c** Cell viability was suppressed by miR-1825 mimic and promoted by miR-1825 inhibitor in A172 cells. **d** Cell migration was suppressed by miR-1825 mimic and promoted by miR-1825 inhibitor in A172 cells. **e** Cell invasion was suppressed by miR-1825 mimic and facilitated by miR-1825 inhibitor in A172 cells. *P < 0.05, **P < 0.01

The role of miR-1825 in GBM was investigated by examining the levels of miR-1825 in GBM cell lines (U251, U87, and A172) and normal human astrocytes (NHA) by qRT-PCR. As shown in Fig. 2a, the expression level of miR-1825 was reduced in all GBM cell lines was significantly lower than that in NHA cell line. We then selected A172 cells for the following experiments. To see miR-1825 role in GBM cell viability, invasiveness, and metastasis, miR-1825 expression was increased or decreased by mimic or inhibitor. As we saw in Fig. 2b, results showed that expression levels of miR-1825 were significantly upregulated by miR-1825 mimic but reduced by miR-1825 inhibitor. Next, MTT assay was carried out for testing A172 cell viability. The findings displayed that increasing miR-1825 inhibited while decreasing miR-1825 enhanced GBM cell viability (Fig. 2c). Transwell assay revealed that A172 cell migration was reduced by miR-1825 mimic but enhanced by miR-1825 inhibitor (Fig. 2c). For invasion, upregulating of miR-1825 expression inhibited A172 cell invasion, while downregulating of miR-1825 expression enhanced A172 cell invasion (Fig. 2d). The above findings indicated that miR-1825 exhibited hindrance effect on cell proliferation, invasion, and migration.

### MiR-1825 upregulation blocked tumor growth in vivo

**Fig. 3:**
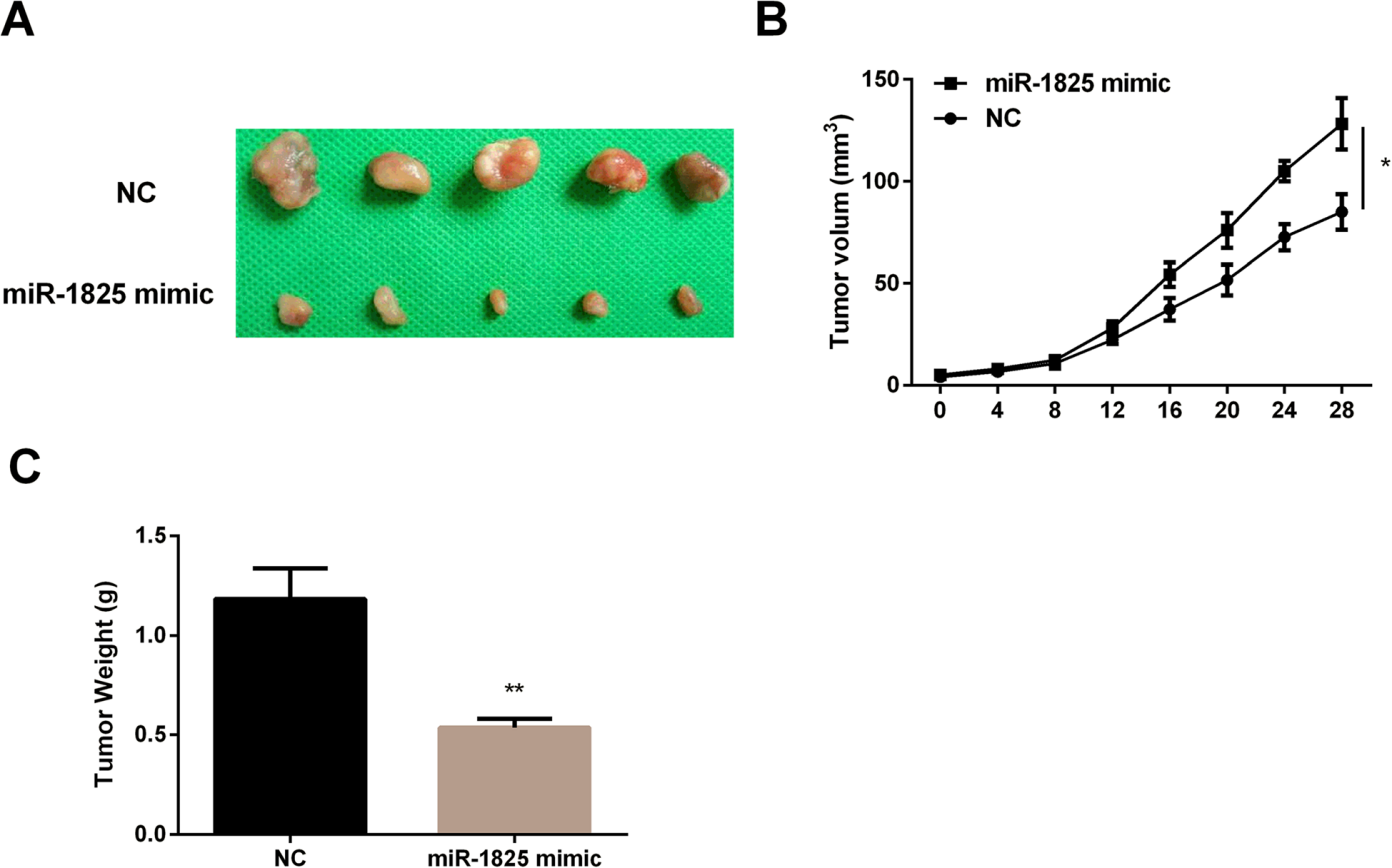
Inhibition effect of miR-1825 on tumor growth. **a** The representative pictures of the size of the tumors in mice transfected with miR-1825 mimic or normal control (NC). **b** The slow growth of tumors in the miR-1825 mimic group compared to that in the NC group. **c** The weight-loss tumors in mice treated with miR-1825 mimic compared to NC. *P < 0.05, **P < 0.01

Then we tested miR-1825 effect on the size and weight of tumors extracted from GBM mice. As we saw in Fig. 3a, the tumor size in miR-1825 mimic group was smaller than that in NC group. Also, miR-1825 mimic made the tumor growth rate slower than the normal control (Fig. 3b). Moreover, miR-1825 mimic reduced the tumor weight in comparison with the NC group (Fig. 3c). All the results demonstrated that miR-1825 upregulation suppressed tumor growth.

### MiR-1825 negatively modulated CDK14 expression

**Fig. 4:**
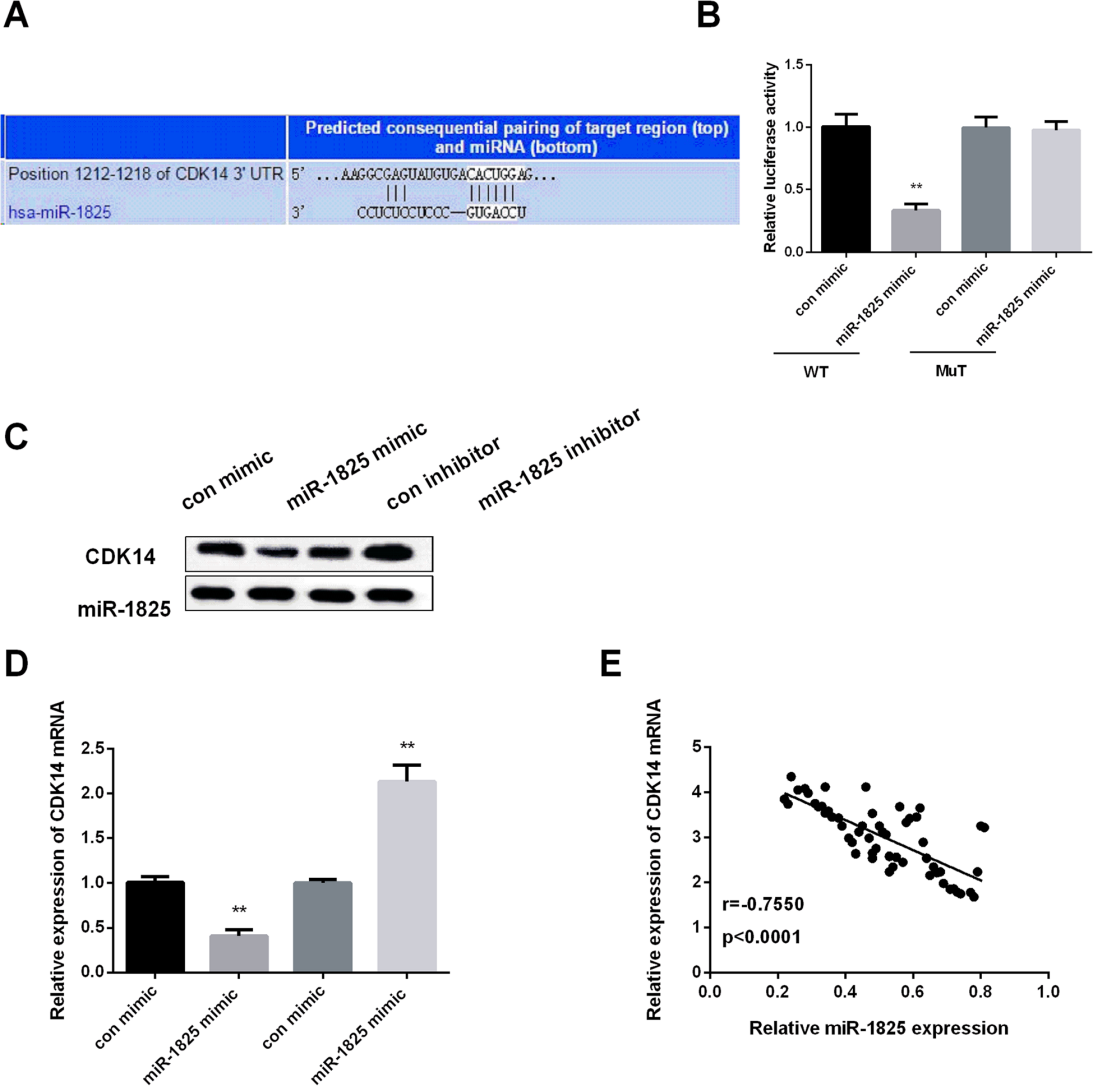
Negatively regulation of CDK14 by miR-1825. **a** Predicted binding site between miR-1825 and 3′-UTR of CDK14. **b** Luciferase activity in A172 cells transfected with WT or MuT CDK14 and miR-1825 mimic or con mimic. **c** Decreased CDK14 level by miR-1825 mimic and increased CDK14 level by miR-1825 inhibitor. **d** Decreased CDK14 mRNA expression by miR-1825 mimic and increased CDK14 mRNA expression by miR-1825 inhibitor. **e** Negatively relationship between CDK14 and miR-1825 in GBM tissue (*r* = −0.7550, *P* < 0.0001). **P < 0.01

As TargetScan prediction, CDK14 was the possible target of miR-1825 and Fig. 4a displayed the binding sides of miR-1825 with CDK14. Furthermore, luciferase reporter assay was applied for further confirming CDK14 was the target of miR-1825. As Fig. 4b displayed that overexpression of miR-1825 significantly suppressed firefly luciferase reporter activity of the WT-CDK14 3′-UTR; however, they did not affect the luciferase activity of MuT-CDK14 3′-UTR. Next, qRT-PCR and Western blot analyses were carried out for detecting the effect of miR-1825 on CDK14 expression in mRNA and the protein level, respectively The findings exerted that miR-1825 mimic exhibited a reduced expression of CDK14, while miR-1825 inhibitor displayed a raised CDK14 expression (Fig. 4c, d). Due to the opposite expression of miR-1825 and CDK14 in GBM, we detected the correlation between miR-1825 and CDK14. Results showed that their relationship was negative (Fig. 4e). These results indicated that CDK14 was the target of miR-1825.

### CDK14 upregulation was associated with poor prognosis

**Fig. 5:**
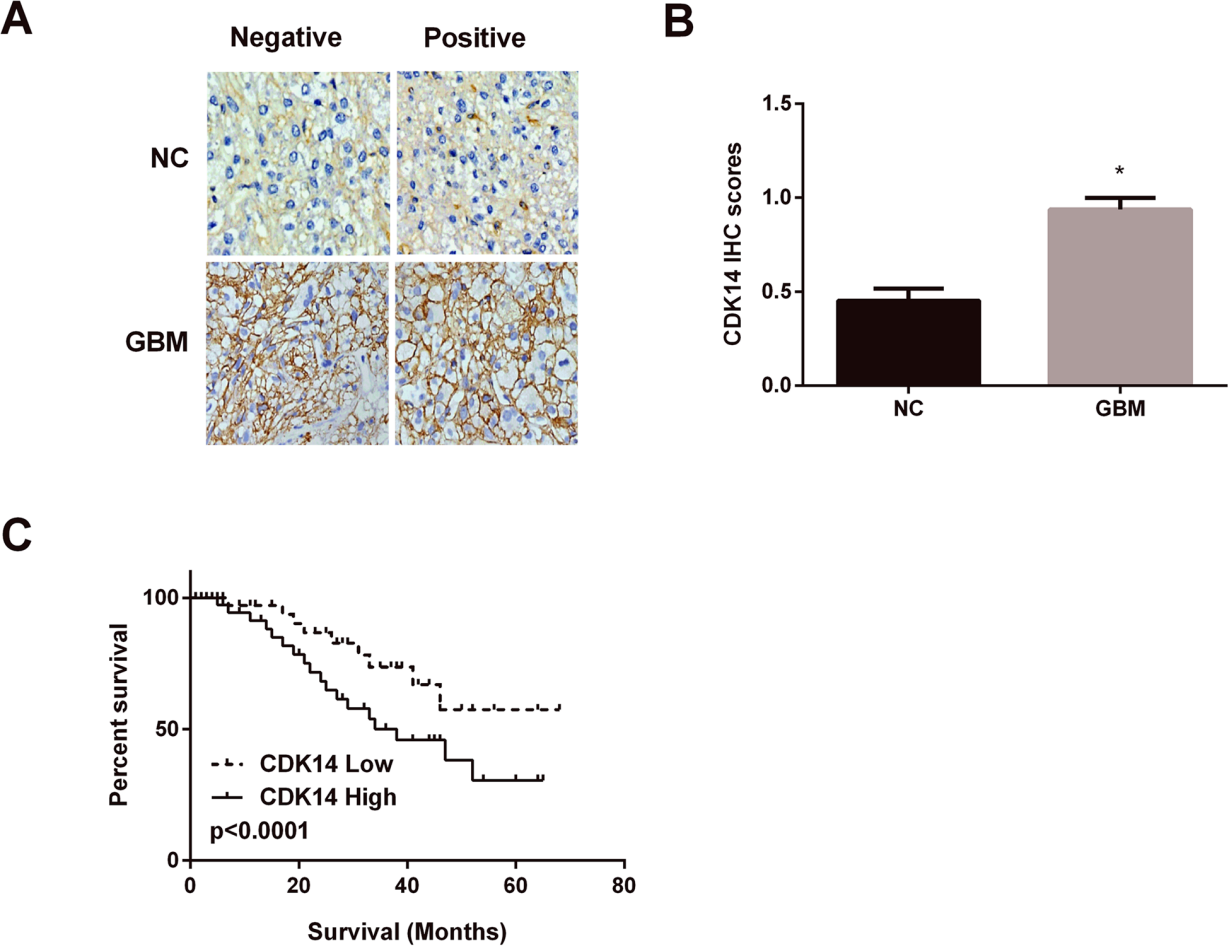
The association of CDK14 differential expression with overall survival. **a** Location of CDK14 in the cell of GBM tissues. **b** Increased protein density of CDK14 in GBM tissues. **c** Low expression of CDK14 in GBM patients exhibited high survival rate of GBM patients. *P < 0.05

We then checked CDK14 expression in GBM tissue samples. The findings displayed that CDK14 was located in cell membrane (Fig. 5a) and its protein density was raised in GBM tissues (Fig. 5b). Moreover, we investigated whether the differential expression of CDK14 was related to patients’ survival rate. The results of Fig. 5c display that the low expression of CDK14 in GBM tissues predicted better prognosis, while high expression of CDK14 predicted worse prognosis. The findings demonstrated that CDK14 upregulation served as an indicator of poorer prognosis in GBM patients.

### MiR-1825 upregulation blocked EMT and Wnt/β-catenin signaling pathway

**Fig. 6:**
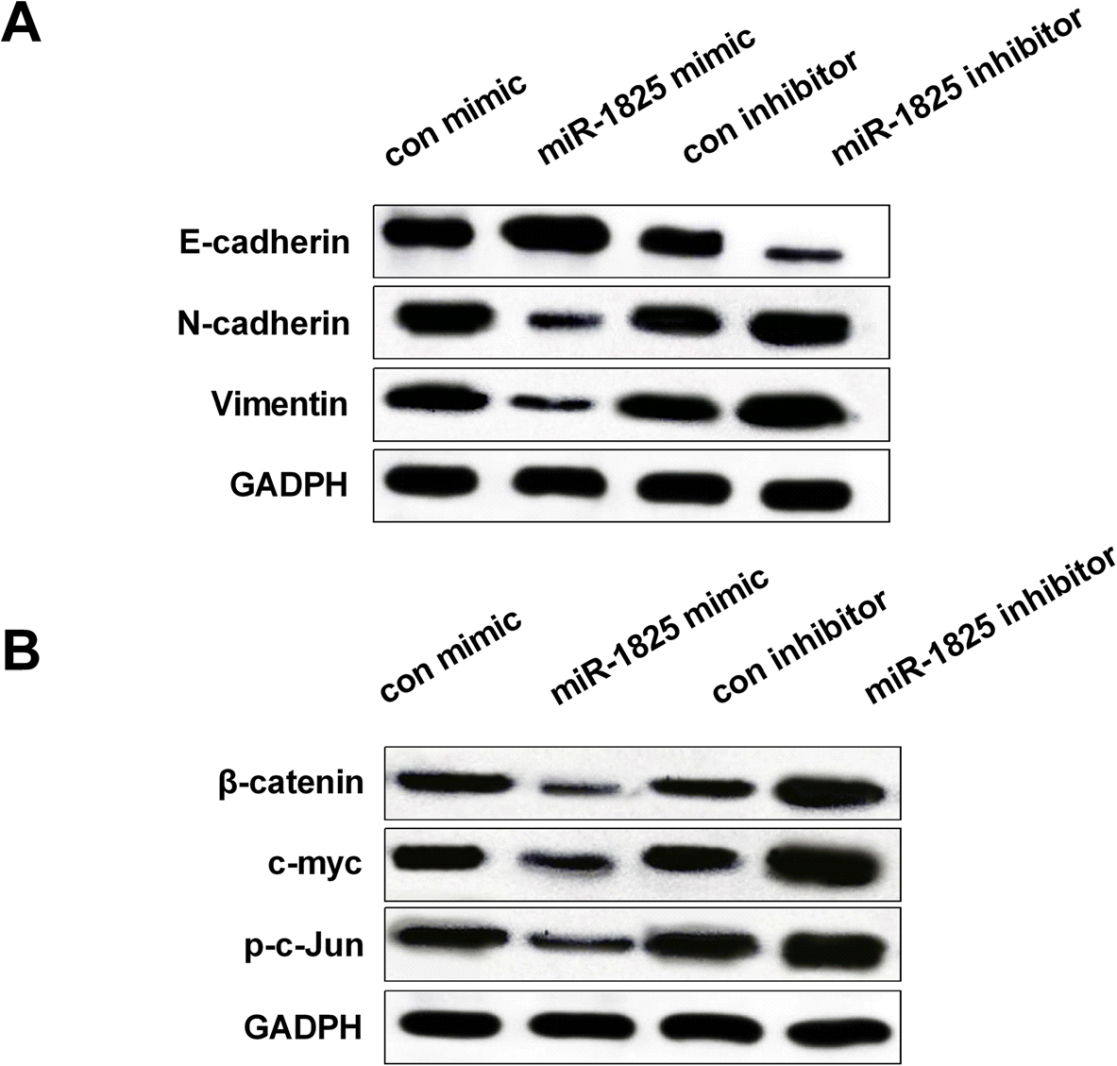
The activation of EMT and Wnt/β-catenin signaling pathway by miR-1825 downregulation. **a** Decreased expression of E-cadherin expression, but increased N-cadherin and vimentin expression by miR-1825 mimic. Increased E-cadherin expression, but decreased N-cadherin and vimentin expression by miR-1825 inhibitor. **b** Reduced β-catenin, c-myc, and p-c-Jun expression by miR-1825 mimic and increased expression of β-catenin, c-myc, and p-c-Jun expression by miR-1825 inhibitor

To further examine the mechanism of miR-1825 in modulating GBM progression, the expression of the downstream genes of EMT and Wnt/β-catenin pathway was tested by Western blot. The findings showed that miR-1825 upregulation enhanced E-cadherin expression, but inhibited N-cadherin and Vimentin expression. However, miR-1825 downregulation displayed the opposite effect (Fig. 6a). As shown in Fig. 6b, increasing miR-1825 reduced β-catenin, c-myc, and p-c-Jun expression, while decreasing miR-1825 exhibited the opposite effect on these levels. The results demonstrated that miR-1825 upregulation blocked Wnt/β-catenin signaling pathway in GBM cells.

## Discussion

Glioblastoma is the most common type of malignant primary intracranial tumor and is characterized by the hallmarks of cellular heterogeneity, rapid proliferation, angiogenesis, extensive invasion, hypoxia, and necrosis [24, 25]. It is an urgent need to gain deeper understanding of molecular mechanisms implicated in glioblastoma progression and development [26]. Many studies have revealed that miRNAs affect GBM development, including miR-146b, miR-148a, and miR-34a [27–29]. These outcomes have attracted in-depth research into miRNAs in GBM.

Previous study has shown that miR-1825 may play an important role in the development of human glioma including apoptosis, cell proliferation, and invasion by microRNA-Gene Ontology network [13]. Here, in our study, we revealed that miR-1825 expression was declined in GBM tissue specimens and its downregulation predicted poor prognosis. However, we also found that CDK14 expression was raised in GBM tissues and its upregulation predicted poor prognosis. Moreover, increasing miR-1825 impeded tumor growth, GBM cell proliferation, invasion, and migration. MiR-1825 negatively regulated the CDK14 expression and Wnt/β-catenin axis.

Researches on miR-1825 in tumors revealed that it served as a potential biomarker in multiple cancers. For example, miR-1825 was highly expressed in prostate cancer and it acted as a prostate cancer biomarker [30]. Also, miR-1825 was upregulated in larynx cancer and involved in tumor progression [31]. However, miR-1825 was downregulated in glioma and associated with tumorigenesis [13]. In this study, we revealed that miR-1825 expression was declined in GBM and used as a predictor for prognosis. Moreover, re-expression of miR-1825 impeded GBM cell viability, invasiveness, and metastasis, which is in line with the research that miR-1825 played an important role in glioma cell proliferation, apoptosis, and invasion [13].

CDK14 was used as an oncogene in several cancers. For instance, its expression was raised in hepatocellular carcinoma as a target of miR-1202 [32]. Besides, CDK14 expression was higher in glioma than normal and took part in glioma progression regulated by miR-613 [20]. All these results supported our above research. Moreover, we first time revealed that CDK14 was the direct target of miR-1825. We also revealed that CDK14 high expression was associated with the poor survival time of GBM patients. Wnt/β-catenin pathway played important roles in tumorigenesis, including GBM. Lots of studies displayed that miRNAs regulated GBM development by modulating Wnt/β-catenin signaling pathway, such as miR-34a [33], miR-328 [34], and miR-21 [35]. Here, we displayed that miR-1825 repressed GBM progression by inhibiting the activation of Wnt/β-catenin signaling pathway. Furthermore, certain reports have suggested that CDK14can regulates a number of pathways, including the Wnt/β-catenin signaling pathway and phosphoinositide 3 kinase (PI3K)/Akt signaling pathway, and cellular mechanisms to act as an oncogene [36, 37]. In the absence of Wnt signaling, the mitosis-specific CDK14-Cyclin Y kinase complex phosphorylates Ser-1490 of LRP5/6 are co-receptors for Wnt ligands at the G2/M stage, thereby triggering the receptor for Wnt-induced phosphorylation [38, 39]. The above studies showed that there is a connection between the regulation of CDK14 and Wnt signaling. We will show the effect of CDK14 on GBM progression and Wnt/β-catenin pathway in our further study. In conclusion, miR-1825 was underexpressed in GBM and suppressed GBM progression, whereas CDK14 was overexpressed in GBM. Moreover, CDK14 was shown as the direct target of miR-1825 in GBM. MiR-1825 upregulation blocked the EMT and Wnt/β-catenin signaling pathway.

## Conclusions

In conclusion, the identification of miR-1825 as a tumor suppressive miRNA in human GBM that acts by targeting CDK14 provides additional evidence of a pivotal role for miRNAs in GBM progression. Given that miR-1825 is downregulated in GBM, the introduction of this mature miRNA into the tumor tissue could serve as a therapeutic strategy by regulating the expression of target genes. MiRNA-based therapeutic patterns are still in their infancy; however, our findings are encouraging and suggest that this miRNA could be targeted for the development of a treatment for patients with GBM in the future.

## Supplementary information

**Additional file 1: Table S1.** Primer sequences for RT-PCR.

## Data Availability

The datasets used and/or analyzed during the present study are available from the corresponding author on reasonable request.
