# Supplementary material for: Upregulation of miR-1825 inhibits the progression of glioblastoma by suppressing CDK14 though Wnt/β-catenin signaling pathway
Source: World J Surg Oncol. 2020 Jun 30;18:147. doi: 10.1186/s12957-020-01927-3 (PMC7325653; doi:10.1186/s12957-020-01927-3)
Supplement: Supplementary file 1 — Additional file 1: Table S1. Primer sequences for RT-PCR. [file 12957_2020_1927_MOESM1_ESM.doc]

***Supplemental Table 1*. Primer sequences for RT-PCR**

| Gene | Primer sequences |
| --- | --- |
| GAPDH | Forward primer: 5′-TATCGGACGCCTGGTTAC-3′  Reverse primer: 5′-CTGGACTGTGAGTACCAGGC-3′ |
| U6 | Forward primer: 5′-CTCGCTTCGGCAGCACA-3′  Reverse primer: 5′-AACGCTTCACGAATTTGCGT-3′ |
| miR-1825 | Forward primer: 5'-TCCAGTGCCCTCCTCT-3'  Reverse primer: 5′-GTCGTATCCAGTGCAGGGTC-3′ |
| CDK14 | Forward primer: 5′- AAGACATCCAACAGCCAGAA-3′  Reverse primer: 5′- TGTAGCCACGTCCCGAGT-3′ |
